# Associations Between Uncertainty Stress, Life Stress and Internet Addiction Among Medical Students

**DOI:** 10.3389/fpubh.2021.809484

**Published:** 2022-01-24

**Authors:** Qian Yang, Zhihua Wu, Xiaozhao Yang, Shuhan Jiang, Dan Wu, John L. Oliffe

**Affiliations:** ^1^Center for Health Policy Studies, School of Public Health, The Children's Hospital, National Clinical Research Center for Child Health, Zhejiang University School of Medicine, Hangzhou, China; ^2^Department of Social Medicine, School of Public Health, Zhejiang University School of Medicine, Hangzhou, China; ^3^School of Sociology and Anthropology, Sun Yat-sen University, Guangzhou, China; ^4^School of Psychology, Shenzhen University, Shenzhen, China; ^5^School of Nursing, University of British Columbia, Vancouver, BC, Canada

**Keywords:** internet addiction, uncertainty stress, life stress, regional economic status, medical college students

## Abstract

**Objectives:**

Internet Addiction (IA) is a growing issue predominate in adolescents and young adults. Although the effects of diverse stressors on IA have been highlighted, there is little consensus about the specific underpinnings of IA. The current study aims to investigate associations between uncertainty stress, life stress and IA among Chinese university medical students.

**Methods:**

A cross-sectional survey employing multi-stage sampling was used. Data were collected from 6,061 students from 27 university medical programs across China. Associations between uncertainty stress, life stress, and IA were examined by means of multivariate logistic regression.

**Results:**

The findings indicated that the overall IA prevalence was 12.6% (95% CI 11.7-13.5), life stress prevalence was 8.1% (95% CI 7.4-8.8), and uncertainty stress prevalence was 19.1% (95% CI 18.1-20.1). Multivariate logistic regression showed that uncertainty stress [adjusted OR 2.60 (95% CI 2.14-3.15), *P* < 0.001] and life stress [adjusted OR 1.71 (95% CI 1.32-2.23), *P* < 0.001] were positively associated with IA. Population Attributable Risk (PAR) of uncertainty stress associated with IA was 29%, and that of life stress was 15%.

**Conclusions:**

The contribution of uncertainty stress to IA is significantly higher than that of life stress. High uncertainty stress, being male and born in a region of higher economic status were associated with excessive Internet use and IA.

## Introduction

Statistics from the Chinese Internet Network Information Center indicate that there are 960 million internet users in China, one-fifth of the world's total (1). The high and rising internet usage has boosted gross domestic product (GDP) and economic development whilst bringing dramatic lifestyle changes that have influenced the emergence of Internet addiction (IA) (2). IA refers to the lack of control over Internet use and can include addiction to online games and use of virtual social networks.

IA has a great impact on student's physical, psychological and social performance. In terms of physical health, it can lead to a range of problem behaviors, such as social problems (3), eating disorders (4), sleep disorders (5, 6), risky sex (7), and even suicide (8). A meta-analysis confirmed that Problematic Internet Use (PIU) is a predictor of eating disorders in students (9). In terms of mental health, internet addiction can increase negative emotions and low happiness (10). Many serious mental illnesses have also been linked to Internet addiction, such as bipolar disorder, social anxiety disorder, and severe depression (11). In addition, in terms of social performance, students with Internet addiction are unable to concentrate on class, truancy and academic performance decline (12, 13). Moreover, they seldom participate in actual social activities, which affects normal interpersonal relationships (3).

IA is increasing in Chinese college students, with addiction rates between 8.12 and 12.83% (14). Chinese medical students have a higher prevalence of IA compared with the other college students, and five times that of the general population (15).

Medical schools are typically competitive environments with prevailing pressures on students (16). The fierce competition for grades can decrease peer support and leave little time or energy for establishing collaborative relationships or meaningful social connections (17). The Association of American Medical Colleges (AAMC) indicated that managing such stresses among medical students is a central component of improving the quality of future medical care (18). In China, the stress of medical workers has also increased (19) with many Chinese doctors expressing reluctance for their children pursue a career in medicine due to such potential occupational dangers and demands (20).

Stress is a major risk factor for addiction behaviors. The uptake of many types of addictive behaviors is a result of a compensatory mechanism that temporarily relieves stress. Research suggests that stressful life events can exacerbate mild to severe IA among college students (21). Differentiating life stress as a general state of concern, uncertainty stress is characterized by anxiety in facing ambiguous situations and problematic environments (22). High life stress levels have also been shown to be predictive of IA for pornography use (23). In a German study of IA, spikes in internet usage were highlighted as an inadequate stress coping strategy (24). While these studies were focused on life stress and IA, uncertainty stress has drawn less attention. However, strong evidence reveals that uncertainty is an influential foundation of stress (25). Previous research has shown that stress from uncertainty can affect the health of medical students. Uncertainty stress of Chinese medical students was associated with a short 27.4% (95% CI 23.9%-30.8%) and long-term illness prevalence 20.0% (95% CI 17.0-23.1%) (26). Besides, as an important variable correlated with mood and social connectedness, uncertainty stress is a critical issue for IA research.

To better understand the associations between stress and IA, we hypothesis that uncertainty stress and life stress would have an impact on IA among Chinese University medical students, and compared the impact of these two pressures on IA.

## Methods

### Study Area and Participants

The current study employed a multi-stage sampling design. In Stage 1, a total of 60 Chinese medical universities were differentiated by regional location. Seven provinces were randomly selected; Jilin, Beijing, Anhui, Shanghai, Zhejiang, Fujian, and Guangdong. Four provinces in the Center; Hubei, Shanxi, Hunan, and Jiangxi. Three provinces in the West; Sichuan, Shanxi, and Tibet. In stage 2, we selected one or two cities as the recruitment sites according to the distribution and number of medical universities in each province. Finally, twenty-seven colleges accepted invitations to participate in this study.

### Data Collection

Based on the multi-stage sampling method, a total of 6,061 students from 27 universities across China were investigated. Of the 27 universities, 11 were in the west, 7 in the middle, and 9 in the east. A standardized questionnaire was administered privately to respondents in schools during regular lectures and class sessions. The inclusion criteria were that respondents learn medical knowledge and skills from the first year of their university studies. Informed consent was received before the respondents began the questionnaire. Ethical approval of the study was obtained from the Human Subjects Ethics Committee of the first authors' institution. All data collected in the study were kept strictly confidential and analyzed in an aggregated manner.

### Measures

Sociodemographic data. Demographic measures included age, sex, and monthly expenses. Monthly expenses (in RMB, the abbreviated form of currency in China) was measured through a question: “How much money do you spend daily each month?” Both the school location and students' birthplace were coded according to the three demarcations of regional economic status. The three regions comprised the following sub-samples; eastern birthplace (68.7%) and school location (53.4%), central birthplace (20.2%) and school location (17.6%) and Western birthplace (11%) and school location (29.0%).

#### Stress (Independent Variables)

The independent variables were life stress and uncertainty stress, measured separately using standard questionnaires designed by Yang et al., which had good reliability and validity (27, 28). Items to evaluate uncertainty stress levels: “Life is subtle, fate is unpredictable,” “The world changes too fast to keep up with it,” “Don't' know what to do to realize my goal,” and “Values of chaos and disorientation.” (α = 0.81) Life stress items included: “Not interested in the professional courses; Feeling bored,” “Learning tasks are heavy,” “Poor learning and living environment,” and “Strained relationships with classmates.” Respondents indicated their stress levels with a five-point scale; “no stress” (1); “little stress” (2); “some stress” (3); “considerable stress” (4); and “excessive stress” (5). A mean score above 3 on any item signified higher stress (28).

#### Internet Addiction (Dependent Variable)

The Internet Addiction Diagnostic Questionnaire (IADQ) outcome variable was adapted from the pathological gambling instrument to evaluate respondents' IA over a 6-months (29, 30). The cutoff score of “five” (of the eight items) was used to differentiate IA. It has been widely employed in China with excellent psychosomatics qualities (31, 32). Yes/No responses were solicited for the eight items.

### Analyses

All data were entered into a database using EpiData 3.1, and entry reliability was assured by double data entry. All statistical analyses were performed using SPSS26.0. We conducted descriptive analyses for all sociodemographic, stress, and internet addiction variables. For the categorical variables, numbers and percentages were used. Besides, we described the prevalence of IA in different gender, age, and other sociodemographic characteristics. Then, the Chi-square test was used to compare the differences of IA rate under different sociodemographic characteristics. Moreover, after controlling for the above sociodemographic characteristics, we conducted multivariate logistic regression to analyze the influence of uncertainty stress and life stress on IA. To determine the contribution of uncertainty stress and life stress to IA, we use the population attributable risk (PAR) statistical analyses. Population attributable risk (PAR) is the proportion of the incidence of disease in the population (exposed and unexposed) due to exposure. It is the incidence of disease in the population that would be eliminated if exposure were eliminated. It measures the potential impact of control measures in a population and is relevant to decisions in public health.

## Results

The mean age of the respondents was 21.79 years old. 95.5% of the respondents were *han* (China's main nationality). Life stress was found in 8.1% (number of people was 5,993), while uncertainty stress was present in 19.1% (number of people was 6,018) of respondents, and 12.6% indicated IA (number of people was 5,644), with significant sex differences wherein 19.2% of males compared to 8.8% of the females reported IA (χ^2^ = 108.88, *P* < 0.001). In addition, different monthly expenses (χ^2^ = 26.14, *P* < 0.001) and birthplace (χ^2^ = 7.74, *P* = 0.02), there are statistical differences in the IA prevalence of students. Descriptive statistics and Chi-square test for IA prevalence of different sociodemographic characteristics are shown in Table 1.

**Table 1:** Descriptive statistics and Chi-square test for IA prevalence of different sociodemographic characteristics (*N* = 5,644).

**Demographic characteristics**	***n***	**% of sample**	**Prevalence (%)**	**adjusted Rao-Scott ***χ^2^*****	***P***
Gender				108.88	<0.001
Male	2,057	36.4	19.2		
Female	3,587	63.6	8.8		
Age (Year)				6.78	0.15
≤ 20	1,201	21.3	11.1		
21	1,165	20.6	13.1		
22	1,402	24.8	14.1		
23	1,208	21.4	12.1		
≥24	668	11.8	12.1		
Monthly expense (Yuan)				26.14	<0.001
<500	721	13.0	9.1		
500~	2,833	51.1	11.1		
1,000~	1,584	28.6	15.1		
1,500~	260	4.7	16.1		
≥2,000	145	2.6	16.1		
Birthplace region				7.74	0.02
West	607	11.0	14.1		
Center	1,113	20.2	10.1		
East	3,780	68.7	13.1		
School province region				2.06	0.38
West	1,473	29.0	13.1		
Center	894	17.6	11.1		
East	2,713	53.4	13.1		

*Missing data exited in each category*.

Multivariate logistic regression analysis with the Enter procedure was employed to assess the effects of gender, birthplace region, monthly expense, uncertainty stress and life stress on IA. Due to the missing values of gender, birthplace region, monthly expense, uncertainty stress and life stress, the logistic regression sample size analysis was 5,359. Finally, the logistic model was statistically significant (χ^2^ = 282.76, *P* < 0.001), and the Hosmer-Lemeshow goodness-of-fit test yielded a *p*-value > 0.05 (*P* = 0.63). Besides, results showed that students born in a region of higher economic status were associated with higher odds of having IA, with stresses having discrepant effects on IA (as shown in Table 2). As expected, the uncertainty stress increased the odds of IA by more than double [OR = 2.60 (95% CI 2.14–3.15)]. However, life stress had a weaker association with IA [OR = 1.71 (95% CI 1.32-2.23)]. PAR of uncertainty stress associated with IA was significantly higher (29%) and that of life stress (15%). Finally, we have obtained the IA prediction equation as below:


$$\begin{array}{l} \text{logit IA =}-\text{3}.\text{01}\beta_{\text{0}}\text{ + 0}.\text{85}\;\text{gender}\\ \text{ + 0}.\text{28}\;\text{birthplace region }(\text{2})\\ \text{ +0}.\text{57}\;\text{monthly expense }(\text{2})\\ \text{ +0}.\text{95}\;\text{Uncertainty Stress + 0}.\text{54 Life Stress} \end{array}$$

birthplace region (2): the participants' birthplace was east (The reference is “Center”), monthly expense (2): the participants' monthly expense was 1,000~ (The reference is “ <500”).

**Table 2:** Multivariate logistic regression analysis of the odds of IA by stress type and selected sociodemographic characteristics, Chinese medical students (*N* = 5,359).

**Demographic characteristic**	***n***	**% of sample**	**Prevalence (%)**		**Internet addiction**		
				**B**	**Unadjusted OR (95% CI)**	**Adjusted OR (95% CI)**	**PAR (95% CI)**
Gender							
Female	3,417	63.8	8.8	0	1	1	0
Male	1,942	36.2	19.2	0.85	2.47 (2.10,2.91)^***^	2.35 (1.98,2.78)^***^	0.20 (0.16,0.25)
Birthplace region							
Center	1,091	20.4	10.1	0	1	1	0
West	600	11.2	14.2	0.31	1.47 (1.09,1.99)^*^	1.36 (1.00,1.87)	0.04 (0.00,0.11)
East	3,668	68.4	13.0	0.28	1.33 (1.07,1.66)^*^	1.31 (1.05,1.65)^*^	0.03 (0.01,0.08)
Monthly expense (Yuan)							
<500	1,538	28.7	10.1	0	1	1	0
500~	3,225	60.2	12.9	0.16	1.32 (1.08,1.60)^**^	1.17 (0.96,1.44)	0.02 (−0.01,0.05)
1,000~	456	8.5	17.3	0.57	1.87 (1.39,2.51)^***^	1.77 (1.31,2.41)^***^	0.12 (0.05,0.20)
1,500~	78	1.5	17.9	0.28	1.95 (1.07,3.56)	1.32 (0.70,2.50)	0.05 (−0.06,0.21)
≥2,000	62	1.2	12.9	0.17	1.32 (0.62,2.83)	1.18 (0.54,2.61)	1.02 (−0.06,0.17)
Uncertainty stress							
Low	4,356	81.3	11.4	0	1	1	0
High	1,003	18.7	26.0	0.95	2.98 (2.50,3.55)^***^	2.60 (2.14,3.15)^***^	0.29 (0.23,0.36)
Life stress							
Low	4,936	92.1	9.8	0	1	1	0
High	423	7.9	24.4	0.54	2.74 (2.17,3.47)^***^	1.71 (1.32,2.23)^***^	0.15 (0.07,0.23)

*All ORs were adjusted by gender, monthly expense and birthplace region, which are significantly associated with IA in Table 2*.

* *P < 0.05*,

** *P < 0.01*,

*** *P < 0.001*.

*Considering the different types of stress in population, we have got the risk ratio in population (PAR) through PAR = P(OR – 1)/[P(OR – 1) + 1]*.

## Discussion

### IA Among University Medical Students

The current study examined associations between uncertainty stress, life stress and IA. Previous studies have established the linkages between life stress and IA (33, 34). Coping strategy and problematic Internet use were predictors of perceived stress in 267 college seniors in the US (34). Turkish research has also demonstrated among college students that stress was positively associated with, and predicted IA (33). Other estimates of IA for European adolescents reported between 1 and 9%, Middle Eastern prevalence was between 1 and 12%, and Asian countries between 2 and 18% (35). The rate of IA in the current sample was 11.8% (95% CI 11.7-12.43%), which falls into the higher levels worldwide and in Asia.

The sex effect confirms previous study findings (36, 37). The sample sex distribution is relatively balanced (63.6% females vs. 36.4% males), *P* < 0.001. The results show the importance of assessing IA risks in Chinese university medical students. In a previous Chines medical student study demographic variable including male, lower grades, one-parent family, and higher monthly expenses were indicated as risk factors for IA (38). These factors are confirmed in other research (39–41).

### Uncertainty Stress, Life Stress, and IA

Though the higher OR of both life stress and uncertainty stress are associated with IA, being male and spending more was also associated with IA. However, PAR of uncertainty stress associated with IA was 25%, that of life stress only was 5%, the former is significantly higher than that of the latter. This means that one in four people's IA was affected by uncertainty stress in the current sample; in contrast, life stress has only a small effect.

Our study supports arguments that stress, especially uncertainty stress acts as a risk factor for IA. Since China has been transitioning from a centralized to a market-based economy, massive social change has occurred along with the erosion of traditional systems. These phenomena provoked considerable uncertainty for residents. As Innes noted, the late-modern experience is the combined outcome of these social forces, giving the sensation of a world that is constantly undergoing change, moving at pace, and based upon a more fluid form of social order. During this epoch, many people feel vulnerable and crave a sense of security and certainty (42).

Our results are consistent with previous research suggesting China's high-stress levels are caused by inequalities and uncertainties embedded in centrally planned and controlled rapid social changes, which in turn impact Chinese peoples' mental health (43) and addiction behaviors (44). Yang et al. have found that uncertainty stress is associated with a range of negative health outcomes, including problematic alcohol use, self-harm, suicidal ideation and poor self-rated health (22). Therefore, uncertainty stress needs more attention.

As the most economically developed region, the (*East*) had better conditions for accessing the internet, which increased the risk for IA. For teenagers in that region, the rapid development of technology increased the ease-of-use for computer technologies; the relatively high GDP also decreased the costs for computer and internet services; and the increasing diversity promoted the availability of internet services, along with social norms around internet usage (45).

PAR of uncertainty stress associated with IA was significantly higher (29%) and that of life stress (15%). Although the life stress is also significantly associated with IA. It is worth noting that the PAR of the former is significantly higher than that of the latter. What's interesting is that we did not observe this for life stress.

### Limitations

Limitations include the cross-sectional survey design. Future work could use follow-up tests and interventions to map changes across time and the life course of respondents who graduate and practice as doctors. That respondents were medical students also limits the generalizability to wider Chinese populations, and college students more generally.

## Conclusions

Uncertainty stress predicted IA among medical college students more significantly than life stress. Being male, richer (which could be seen in the amount they spend every month), and born in a region of higher economic status also heightened the risk for IA prevalence. In conclusion, this study provides preliminary evidence for public health policymakers and educators, and suggests an approach for intervening to prevent or reduce IA among university students. We recommend that IA interventions include management of uncertainty stress, especially targeting males in the East region.

## Data Availability Statement

The raw data supporting the conclusions of this article will be made available by the authors, without undue reservation.

## Ethics Statement

The studies involving human participants were reviewed and approved by Zhejiang University School of Public Health Ethics Committee. The patients/participants provided their written informed consent to participate in this study.

## Author Contributions

QY and XY contributed to the conception of the study. SJ and DW collected the data. QY, ZW, and JO carried out data cleaning. QY and ZW performed the data analyses and wrote the manuscript. XY and JO contributed to critically revising the manuscript for important content. All authors contributed to the article and read and agreed to the published version of the manuscript.

## Funding

This research was funded by National Natural Science Foundation of China (grant number 71974170) and Leading Innovative and Entrepreneur Team Introduction Program of Zhejiang (grant number 2019R01007).

## Conflict of Interest

The authors declare that the research was conducted in the absence of any commercial or financial relationships that could be construed as a potential conflict of interest.

## Publisher's Note

All claims expressed in this article are solely those of the authors and do not necessarily represent those of their affiliated organizations, or those of the publisher, the editors and the reviewers. Any product that may be evaluated in this article, or claim that may be made by its manufacturer, is not guaranteed or endorsed by the publisher.
